# Impact of serial clinical swallow evaluations and feeding interventions on growth and feeding outcomes in children with long-gap esophageal atresia after anastomosis: a retrospective cohort study

**DOI:** 10.1007/s12519-024-00850-x

**Published:** 2024-11-15

**Authors:** Jun-Li Wang, Run-Qi Huang, Chun-Yan Tang, Wen-Jie Wu, Fei Li, Tai Ren, Jun Wang, Wei-Hua Pan

**Affiliations:** 1 Developmental and Behavioral Pediatric Department, Xinhua Hospital Affiliated to Shanghai Jiao Tong University School of Medicine, 1665 Kongjiang Road, Shanghai, 200092 China; 2 Child Primary Care Department & Ministry of Education-Shanghai Key Laboratory of Children’s Environmental Health, Xinhua Hospital Affiliated to Shanghai Jiao Tong University School of Medicine, 1665 Kongjiang Road, Shanghai, 200092 China; 3 Department of Pediatric Surgery, Xinhua Hospital Affiliated to Shanghai Jiao Tong University School of Medicine, 1665 Kongjiang Road, Shanghai, 200092 China

**Keywords:** Clinical swallow evaluation, Feeding disorder, Long-gap esophageal atresia, Nutritional status

## Abstract

**Background:**

Children undergoing surgical anastomosis for long-gap esophageal atresia (LGEA) often suffer from complications related to delayed oral feeding, which may impair their early development. Clinical swallow evaluation (CSE) is an effective technique to improve feeding outcomes. However, there are limited evidences on the application of CSE in these children.

**Methods:**

Since 2020, serial CSEs have been consistently implemented for children undergoing anastomosis for LGEA in our hospital. We conducted a retrospective study comparing 19 children who received CSE with 31 historical controls who did not. Inverse probability of treatment weighting (IPTW) was applied to balance preoperative characteristics. We compared the time from surgery to full oral feeding and the rate of postoperative complications between the two groups. Growth curves for length-for-age *Z* score (LAZ) and weight-for-age *Z* score (WAZ) up to age 3 were fitted using generalized additive mixed models.

**Results:**

The median time to full oral feeding was 1.1 months [interquartile range (IQR), 0.8–2.4] in the CSE group and 1.5 months (IQR, 0.6–5.7) for controls. After IPTW, CSE was associated with a shorter time to full oral feeding, with a weighted hazard ratio of 2.26 [95% confidence interval (CI), 1.21 to 4.24]. LAZ growth curves significantly differed between groups (*P* = 0.001).

**Conclusion:**

CSE was associated with the expedited achievement of full oral feeding and a more favorable growth pattern before 3 years of age.

**Graphical abstract:**

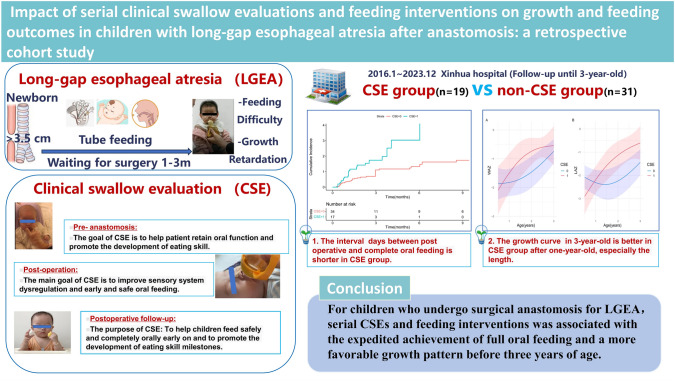

**Supplementary Information:**

The online version contains supplementary material available at 10.1007/s12519-024-00850-x.

## Introduction

Esophageal atresia is a rare congenital malformation, with a reported incidence ranging from 1 in 2500 to 1 in 4500 live births [1, 2]. Over the past few decades, surgical advancements have transformed potentially life-threatening malformations into manageable conditions with favorable prognoses [3]. However, a specific subtype of esophageal atresia, known as long-gap esophageal atresia (LGEA), continues to pose unique challenges because the long gap between the esophageal segments precludes primary anastomosis in the neonatal period [4–6]. In addition to surgical difficulty, managing LGEA requires multiple medical procedures within the first postnatal year, a critical period for developing swallowing function and overall growth in infants. Furthermore, evidence on the optimal perioperative care for infants who undergo surgical anastomosis for LGEA remains limited.

Before oral feeding can be initiated, infants with LGEA must undergo several invasive procedures such as tracheal intubation, esophageal drainage, tube feeding, and tracheal intubation. These procedures are necessary to repair the structural anomalies of the esophagus. However, the prolonged duration of these interventions can result in delayed or limited oral feeding opportunities during critical developmental windows, which may lead to deficiencies in oral feeding [7, 8]. Consequently, initiating oral feeding for children after surgery may be difficult. Delayed oral feeding in early life may further impair dietary intake, as well as the overall growth and development of children. Feeding and swallowing problems are common after surgery for esophageal atresia, and the early involvement of a multidisciplinary team has been recommended to help these patients [9–12]. However, there is still limited data on the outcomes of systematic management approaches for esophageal atresia patients [13].

Clinical swallow evaluation (CSE) is a systematic strategy, proposed by Suitor et al. [14], to evaluate dysphagia and guide individualized intervention (Table 1). This procedure aims to improve the feeding and growth outcomes of children who undergo LGEA surgery. A typical CSE includes five steps: a preassessment case review, a caregiver/child interview, a direct assessment, risk factor screening, and a treatment plan with suggestions [14]. Developmental-behavioral pediatricians perform CSEs to create individualized treatment plans for children and their parents [15]. Since 2020, CSE has been implemented in our hospital in both the pre- and postoperative periods for children undergoing LGEA surgery. In this retrospective cohort study, we included a historical control group and used propensity score-based methods to investigate whether CSE was associated with a shorter period from LGEA surgery to full oral feeding. We also explored whether CSE could improve length and weight growth patterns in children who undergo LGEA surgery.

**Table 1:** Procedure of the clinical swallowing evaluation (CSE) program

Items	Assessment points		Procedures
Evaluation of eating skills	Preassessment case review		Based on the child’s medical history, developmental progress, and feeding experiences, the evaluator should formulate potential clinical hypotheses regarding the child’s eating skill development
	Feeding performance	Caregiver interview Feeding history Current feeding status	Identify any conditions or complications that may disrupt the typical advancement of feeding capabilities Investigate the onset of any feeding challenges to establish if they are enduring or temporary Investigate data regarding the child’s dietary routine, including the timing and setting of meals, targeted approaches utilized, and details of both oral and tube-based nutritional intake
		Direct evaluation Oral examination Feeding skills Instrumental evaluation (optional)	Assess the anatomical and functional integrity of swallowing; discern the underlying causes of the skill deficiencies
Screening for potential risk factors	Common potential risk factors: Swallow function Respiratory system Gastrointestinal/nutritional problems Nervous system problem Developmental problem Other medical problem		Identify potential risk factors for feeding difficulties
Interventions	Consultation, recommendation and treatment plan		1. Offer guidance and education to caregivers regarding the causes, expected outcomes, dietary considerations, proper feeding process, parent–child interaction during meal time, and therapeutic requirements; 2. Formulate a feeding strategy to ensure children consume food safely and efficiently; 3. Establish a therapeutic regimen, outlining targeted objectives and methodologies for dietary habits and skill development; 4. Recommend further evaluations and additional medical consultations as necessary

## Methods

### Study setting and participants

We conducted a retrospective cohort study using data from electronic medical records and follow-up visits. Typically, children were diagnosed with LGEA prenatally or within 1 month after birth and were then referred to the Department of Pediatric Surgery at Xinhua Hospital in Shanghai for treatment. Xinhua Hospital is a tertiary center serving patients nationwide, mainly from the Yangtze Delta region. Given that the reported incidence of LGEA was 1 per 40,000 live births [4], the nine cases of LGEA treated annually in Xinhua Hospital correspond to a population of about 0.36 million, significantly greater than the 0.07 million live births recorded in Shanghai in 2022 [16]. This study involved all children (*n* = 58) who underwent LGEA surgery at Xinhua Hospital between January 2016 and December 2023. The patients were scheduled for outpatient follow-ups after surgery three times before reaching three years of age: at 6 months, 12 months, and between 2 and 3 years of age. We excluded eight children who had only completed one follow-up because of missing data on the main outcome of long-term growth.

The majority of LGEA cases required a delayed primary anastomosis (see LGEA management below), which had been recognized to influence swallow function and associated with delayed oral feeding and growth outcomes. To improve the nutritional and growth outcomes of children who underwent LGEA surgery, a standardized CSE program was introduced in January 2020, in collaboration with the Department of Developmental and Behavioral Pediatrics. Traditionally, patients who experienced an extended duration before achieving full oral feeding were managed with expectant care within the surgical departments. Referrals to developmental pediatricians were typically reserved for cases where oral feeding was not established by 2–3 years of age, specifically when associated with certain conditions such as coughing while drinking water, a restricted diet limited to certain textures, or reliance on tube feeding. This practice continued until the implementation of the CSE program in 2020. Consequently, patients who underwent anastomosis before the introduction of the CSE program did not receive CSE and were thus included in the historical control group (*n* = 31). Conversely, patients who underwent anastomosis after the implementation of the CSE program received CSE and were included in the intervention group (*n* = 19).

### LGEA assessment and management

Esophageal atresia was suspected based on prenatal imaging, which revealed a structure resembling a blind pouch-like shape in the upper esophagus, no stomach bubbles or a small stomach bubble, and a history of increased amniotic fluid after 24 weeks of pregnancy. Esophageal atresia was confirmed via a tube inserted in the baby’s nose or mouth cannot pass down into the stomach, and x-ray can confirm that the tube stops in the upper esophagus. Esophagography was performed to measure the distance between the proximal and distal segments of the esophagus. LGEA was defined as type I, type II, or type IIIa esophageal atresia with a distance between the two segments greater than 3 cm [4]. All patients diagnosed with LGEA underwent gastrostomy via a nutritional route, except for one patient who had a short distance from the blind pouch (3.5 cm) and underwent primary esophageal anastomosis. After gastrostomy, continuous suction in the upper pouch was applied to avoid aspiration pneumonia. A bougienage stretching technique was used in the hospital to shorten the gap between the proximal and distal segments of the esophagus [17]. Once satisfactory elongation was achieved, a tension-free anastomosis was performed via thoracoscopy in 48 of the 50 included children. For the two children whose elongation processes were unsatisfactory, esophageal replacement was performed to construct the esophagus (thoracotomy).

The management protocol for LGEA patients is depicted in Supplementary Fig. 1. The transition from fasting to full oral feeding involves a three-step process: parenteral and tube feeding, partial oral feeding, and full oral feeding. Briefly, after surgery, patients were required to fast until the anastomosis had healed, which was confirmed by esophageal radiography. The first esophageal radiography was arranged 7–10 days post-surgery, with subsequent radiographs conducted weekly if the anastomosis had not healed. Partial oral feeding was initiated once anastomosis healing was confirmed. Tube feeding was discontinued once full oral feeding could be achieved.

### Clinical swallow evaluations

We expanded this framework by integrating multiple pre-, peri-, and post-operative sessions, with each session comprising both evaluative and interventional components, tailored for patients with LGEA who underwent anastomosis (Supplementary Fig. 1). Briefly, a developmental–behavioral pediatrician (“the evaluator” WJL, Supplementary Table 3–5) conducted CSEs via individualized examinations and interventions during the pre-, peri- and post-surgery phases for LGEA patients. The objective was to develop age-appropriate eating skills and improve nutritional status. In this study, all CSE procedures were executed by one developmental-behavioral pediatrician (WJL) following an established protocol. First, the evaluator assessed the eating skills of each child to identify feeding problems that could delay the development of normal eating skills. This assessment included a preassessment case review, a caregiver interview, and a direct assessment. In the direct assessment, the evaluator examined the anatomy of the oral cavity, evaluated oral feeding mechanics including the functions of the lips, cheeks, tongue, and jaw, as well as related feeding proficiencies and assessed the coordination of feeding, swallowing, and breathing by administering 0.5–1 mL of water to infants using a syringe while simultaneously auscultating the neck [18]. Next, the evaluator screened for potential risk factors that could affect feeding difficulties, including swallow function, respiratory system, gastrointestinal/nutritional issues, the nervous system, developmental concerns, and other medical conditions. Then, the evaluator developed individualized therapeutic and feeding plans for patients. The therapeutic plan provided caregivers with advice on skill development and bedside instructions on relevant techniques. It further entailed administering targeted interventions aimed at fostering new skill acquisition, enhancing strength and coordination, and improving the safety of the swallowing process, such as the sequential introduction of liquids and solids, avoiding foods that did not align with the patient’s oral feeding capabilities, and food modifications to facilitate increased consumption. Individualized exercises such as simulated sucking, oral motor training, hand-to-mouth coordination activities, and non-nutritive bottle-sucking practice were also incorporated. Notably, the evaluator imparted essential exercises to the caregivers and offered individualized dietary advice, addressing aspects like food consistency and variety. This protocol guaranteed a regimen of daily practice sessions, occurring three to six times daily for optimal effectiveness.

CSE was systematically scheduled throughout the study’s duration, with a preoperative assessment conducted monthly. Postoperatively, CSE was administered a minimum of three to five times within the first postoperative year, initially at about 7–10 days post-anastomosis, with a subsequent evaluation at the one-month follow-up, and then evaluations every three months thereafter. For patients with esophageal dysfunction that limited their oral feeding capabilities, a re-evaluation was promptly arranged one week after the initial assessment to support the enhancement of the child’s feeding skills. If full oral feeding remained unattainable, monthly CSE re-evaluations were instituted to encourage further development in this area. When safe oral feeding was not feasible, oral exercise guidance was provided, followed by a reassessment three to seven days later, with weekly assessments continuing thereafter. During this period, the nursing team offered daily bedside intervention based on the assessment outcomes and instructed caregivers to facilitate three to six or more practice sessions daily. The protocol continued unchanged upon confirmation of the child’s safe oral feeding ability, with the frequency of evaluations primarily dictated by the developmental milestones of the child’s feeding skills. Patients in the treatment group received a minimum of one preoperative CSE and at least three postoperative CSEs. The evaluator delivered treatment and recommendations at the bedside during the patient’s hospital stay and continued this guidance during subsequent outpatient clinic visits. Notably, the recommendations focused on family empowerment through caregiver education, emphasizing that the intervention exercises were predominantly administered by caregivers both in the hospital and at home (Table 1).

**Fig. 1:**
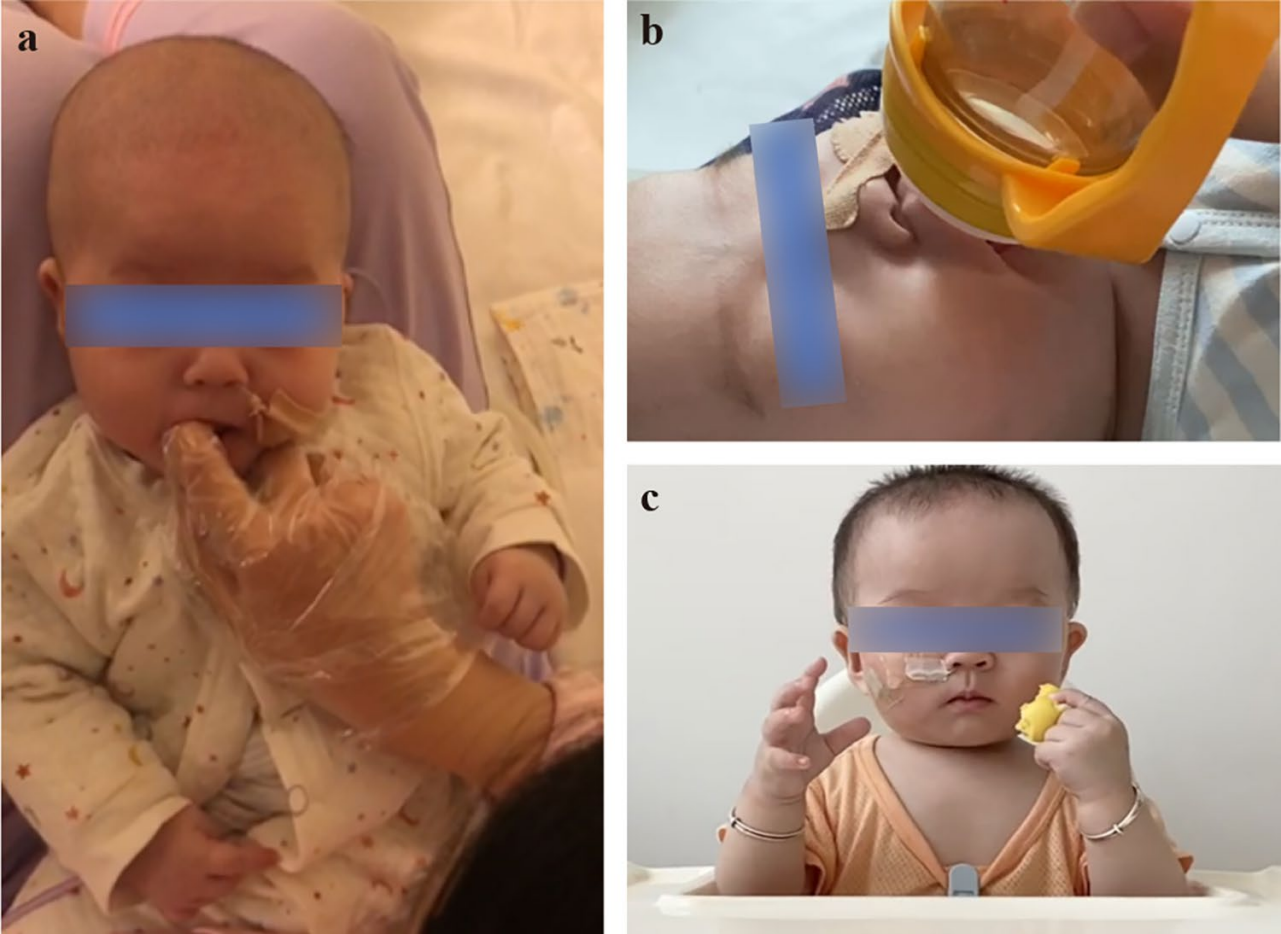
Typical clinical swallow evaluation interventions for a boy diagnosed with long-gap esophageal atresia. **a** before the operation, **b** during the perioperative period, and **c** during the postoperative period

The CSE program for LGEA patients was conducted in conjunction with various departments, including pediatric surgery, intensive care, nursing, pediatric respiratory, pediatric gastroenterology and nutrition, and otolaryngology departments. Referrals to pertinent specialties by the evaluator were made as required to support the patients’ comprehensive care. We present details of the CSE of one child who underwent LGEA surgery for a clearer understanding (Table 2, Fig. 1). The boy was diagnosed with congenital esophageal atresia (type I) through laparoscopic gastrostomy and distal esophagography. The distance between the two ends of the esophagus was 7.5 cm. After several rounds of pre- and postoperative CSE, the boy achieved full oral feeding at 8 months of age, 17 days after the anastomosis.

**Table 2:** A typical case of patient underwent clinical swallowing evaluation (CSE) program

Items	Before the operation (1–7 mon)	Perioperative period (7 mon 7 d–7 mon 23 d)	After the operation (8–24 mon)
Evaluation of eating skills	Parenteral nutrition for nutritional support Enteral nutrition support through gastrostomy (tube feeding)	Enteral nutrition support through gastrostomy (tube feeding)	Maintenance of gastrostomy for a duration of one to two months
	The insertion of a salivary drainage tube into the oral cavity induces a state of oral sensory aversion	Improved oral sensory function Reduced oral suction capacity Delayed motor development	The aim was to preserve oral function
Screening for potential risk factors	Moist sounds in the pharyngeal region, improved by vertical positioning of the head	Inability to sit alone	X-ray imaging to confirm postoperative esophageal function is a prerequisite for safe oral feeding
Intervention	A salivary drainage tube is introduced through the nasal cavity to minimize oral discomfort Exercise through false sucking and oral training Facilitate mother–child dyadic interaction by encouraging eye contact and lip stimulation (Fig. 1a) Prepare for oral feeding Physical rehabilitation to improve trunk stability Regular application of CSE, such as hand-mouth and hand-object-mouth exercises Referral to the otolaryngology department to exclude tracheomalacia	Introduce airless bottle suction practice before surgery to prepare for postoperative oral feeding (Fig. 1b) Referral to a specialized nursing team for caregiver education on gastrostomy care	Prior to oral feeding, the child must practice swallowing water. After swallowing 5 ml of water proficiently, oral feeding of special formula milk is started. In 3 days, full oral feeding is achieved Follow-up at 2 years of age and continue regular CSE to help introduce solid foods, food diversity, and guide responsive feeding to promote early development in children (Fig. 1c)

We report one case to illustrate CSE for patients who undergo surgical anastomosis for long-gap esophageal atresia (Fig. 1). Intrauterine ultrasound at 33 weeks of pregnancy indicated that stomach bubble was not present. At 38 + 3 days of pregnancy, the patient underwent a cesarean section, and the male infant’s birth weight was 2900 g. On the day of birth, congenital esophageal atresia (type I) was diagnosed through laparoscopic gastrostomy and distal esophagography. Given that the distance between the two ends of the esophagus was 7.5 cm, the infant had a longer waiting period for surgery and was tube-fed during this time. Surgical anastomosis was performed at the age of 7 months and 23 days. After several rounds of pre- and postoperative CSE, the infant achieved full oral feeding at 8 months of age, 17 days after undergoing surgical anastomosis

### Full oral feeding, physical measurement, and postoperative complications

We evaluated the feeding and growth status of the children after surgery using two primary outcomes: the period between surgery and full oral feeding, and the growth curve in early life. The time of full oral feeding was defined as the date on which a child demonstrated the ability to coordinate sucking, swallowing, and respiration for safe oral feeding, and tube feeding was discontinued [19]. Data on feeding conditions after surgery were collected weekly during hospitalization and every 3 months during outpatient follow-up. To analyze the period between surgery and full oral feeding, we defined a time-to-event period that started at the date of surgery and ended at the date of transition from tube feeding to full oral feeding, death, or loss to follow-up, whichever occurred first.

Length and weight data were collected through physical examination during outpatient follow-up. We calculated the length-for-age *Z* score (LAZ) and weight-for-age *Z* score (WAZ) using WHO Anthro software (version 3.2.2). We compared group-average growth curves for the WAZ and LAZ. These two growth curves fluctuate around zero for a normally developing child. In addition, we investigated stunting, defined as an LAZ <  −2, and underweight, defined as a WAZ <  −2, at 24 to 36 months of age [20, 21].

We recorded postoperative complications that occurred during hospitalization and outpatient follow-up, including pneumonia, anastomotic stenosis, reflux esophagitis, hiatal hernia, anastomotic leak, tracheo-esophageal fistula, and tracheal stenosis. All the data were extracted from electronic medical records and coded as yes or no during the follow-up period.

### Covariables

We selected potential confounders to establish the propensity score, including sex (male or female), age at surgery (in months), place of residence (urban, rural, or suburban), distance from the blind pouch of the esophagus before stretching (in cm), and weight at the time of surgery [19, 20]. We also included other clinical characteristics, including preterm birth status (yes or no), birth weight, prenatal diagnosis of LGEA, type of surgical procedure (thoracotomy or thoracoscopy), gastrostomy status (yes or no), and the presence of congenital anomalies (yes or no, for each type). Congenital anomalies included the following types: hypothyroidism, cleft palate, urinary system defects, cardiac malformations, skeletal muscle deformities, gastrointestinal malformations, and imperforate anus. All data were collected from electronic medical records.

### Statistical analysis

We applied the inverse probability of treatment weighting (IPTW) approach to account for potential confounding factors. Briefly, a propensity score is the probability of a patient receiving the treatment of interest based on potential confounders (as described above). Logistic regression was applied to calculate the propensity score for each individual, using the *ipwpoint* function from the package ipw (version 1.2). Patients who received CSE were weighted by 1/propensity score and those who were in the reference group were weighted by 1/(1−propensity score). We used the standardized mean difference (SMD) to compare the differences in means (numerical variables) and proportions (categorical variables) of the baseline characteristics between the CSE group and the non-CSE group. Given the small sample size, an SMD < 0.2 was used as the threshold for an acceptable balance.

We performed all the analyses described below after IPTW. To investigate the association between CSE and the period between surgery and full oral feeding, we used the weighted Kaplan‒Meier method for visualization and the Cox proportional hazard model to estimate hazard ratios (HRs) and 95% confidence intervals (CIs). In an additional analysis, we adjusted for the same set of covariates for comparison. Then, we used logistic regression to calculate the odds ratios (ORs) with corresponding 95% CIs for the associations between CSE and stunting at 2–3 years of age, underweight at 2–3 years of age, and postsurgical complications. Children without follow-up data between 2 and 3 years of age were not included in the investigation of stunting and underweight. Since there were no deaths during the follow-up period, we did not use methods considering competing events. We modeled the growth curves for WAZ and LAZ using a restricted cubic spline with three knots on age to account for potentially nonlinear growth patterns in the study period. All analyses were performed using R (version 4.2.1).

## Results

Among the 50 patients who underwent surgical anastomosis for LGEA, 19 (38%) underwent CSE. Compared with historical controls who did not receive CSE, patients who did receive CSE were more likely to live in urban areas, have a lower weight before surgery, and have a longer distance from the blind pouch of the esophagus (Table 3). After IPTW, a satisfactory balance in the distribution of these characteristics was obtained (Table 3, Supplementary Fig. 2, Supplementary Table 1). The birth outcomes, surgical procedures, and congenital anomalies are detailed in Table 3. The mean number of follow-up visits was 3 (SD, 0.5) for patients under 5 years of age. There were no deaths during the follow-up period.

**Table 3:** Baseline characteristics of the eligible children who underwent surgery for long-gap esophageal atresia with and without clinical swallow evaluations (CSE) before and after inverse probability weighting

Characteristics	Study group, unweighted			Study group, IPTW		
	No CSE (*n* = 31)	CSE (*n* = 19)	SMD	No CSE (*n* = 33.8)	CSE (*n* = 16.7)	SMD
Age at surgery, d [mean (SD])]	135.0 (76.6)	127.0 (93.3)	0.09	125.4 (69.5)	121.4 (83.1)	0.05
Sex						
Female	15 (48.4)	8 (42.1)	0.13	15.1 (44.8)	8.9 (53.3)	0.17
Male	16 (51.6)	11 (57.9)		18.6 (55.2)	7.8 (46.7)	
Place of residence			0.30			0.15
Urban	15 (48.4)	12 (63.2)		18.2 (53.9)	7.7 (46.4)	
Rural or suburban	16 (51.6)	7 (36.8)		15.6 (46.1)	8.9 (53.6)	
Weight before surgery, kg [mean (SD])	5.7 (1.9)	5.0 (1.9)	0.36	5.3 (1.7)	5.1 (1.7)	0.11
Distance from the blind pouch of the esophagus, cm [mean (SD])	4.6 (1.3)	5.7 (1.5)	0.82	5.4 (1.9)	5.4 (1.3)	0.05
Preterm birth	15 (48.4)	9 (47.4)	0.02	14.8 (43.8)	7.1 (42.3)	0.03
Birth weight, g [mean (SD])	2686 (491)	2337 (390)	0.79	2553 (478)	2349 (348)	0.49
Prenatal diagnosis of long-gap esophageal atresia	21 (67.7)	12 (63.2)	0.10	23.7 (70.3)	10.6 (63.6)	0.14
Surgical procedure			0.49			0.35
Thoracotomy	0 (0.0)	2 (10.5)		0.0 (0.0)	1.0 (5.8)	
Thoracoscopy	31 (100.0)	17 (89.5)		33.8 (100.0)	15.7 (94.2)	
Gastrostomy	31 (100)	18 (94.7)	0.33	33.8 (100)	15.9 (95.2)	0.32
Congenital anomalies						
Hypothyroidism	0 (0)	1 (5.3)	0.33	0 (0)	1.0 (5.8)	0.35
Cleft palate	0 (0)	1 (5.3)	0.33	0 (0)	1.0 (5.8)	0.35
Urinary system defect	1 (3.2)	2 (10.5)	0.29	0.8 (2.4)	1.3 (7.9)	0.25
Cardiac malformation	5 (16.1)	4 (21.1)	0.13	3.8 (11.1)	4.9 (29.3)	0.47
Skeletal muscle deformity	3 (9.7)	2 (10.5)	0.03	3.2 (9.5)	1.7 (10.3)	0.03
Gastrointestinal malformation	1 (3.2)	2 (10.5)	0.29	1.1 (3.1)	1.1 (6.4)	0.16
Imperforate anus	2 (6.5)	2 (10.5)	0.15	3.9 (11.6)	1.1 (6.4)	0.18

The data are presented as the number (percentage) of patients unless otherwise indicated

*IPTW* inverse probability of treatment weighting, *SMD* standardized mean difference

**Fig. 2:**
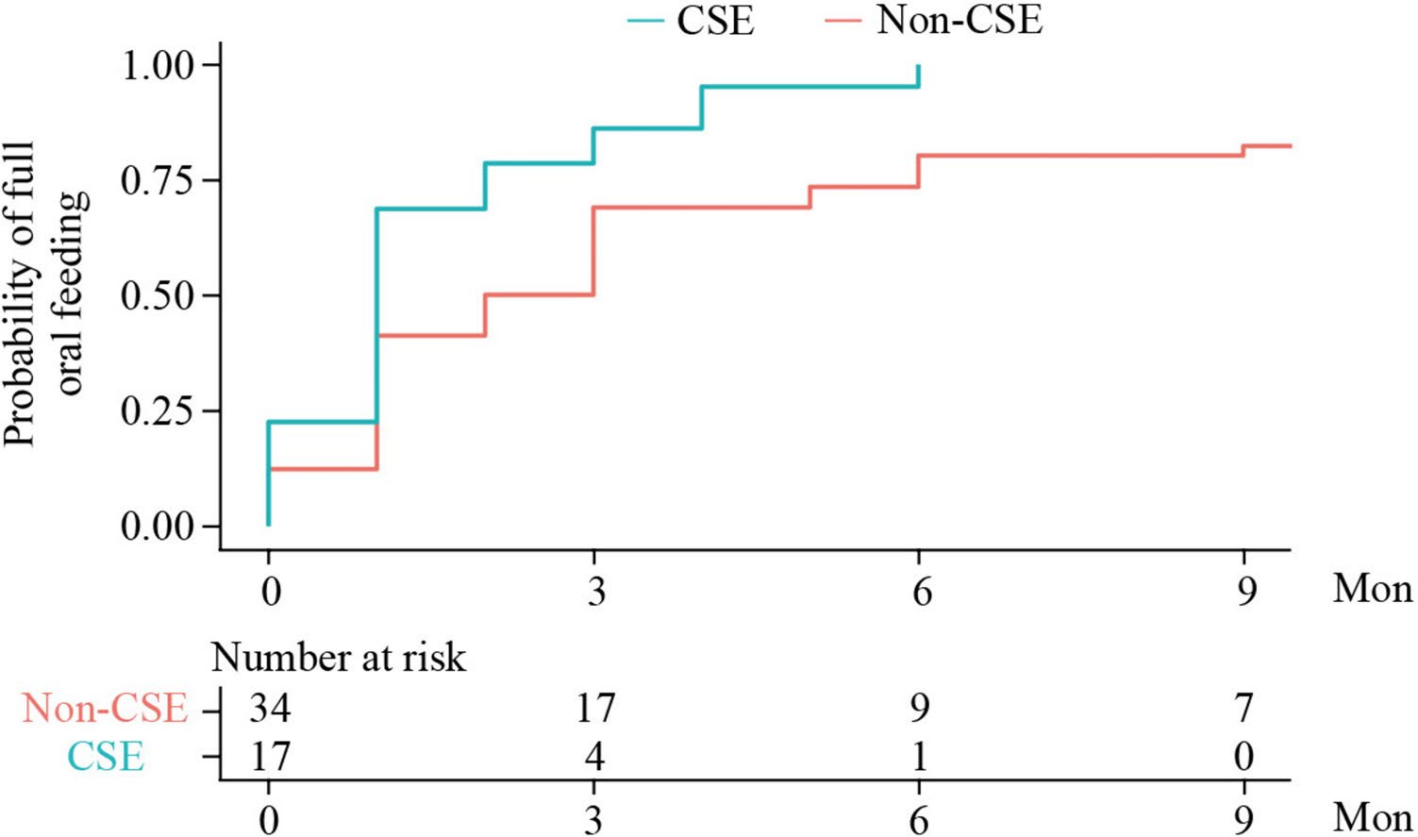
Probability of achieving full oral feeding in children who underwent surgery for long-gap esophageal atresia with and without CSE. The period between surgery and full oral feeding was defined as the time-to-event, with follow-up starting on the date of surgery and the event defined as the date when full oral feeding was achieved. The number at risk refers to the numbers weighted in both the CSE and non-CSE groups after IPTW to account for potential confounders, including age at surgery, sex, place of residence, weight before surgery, and distance to the blind pouch of the esophagus. *CSE* clinical swallow evaluation, *IPTW* inverse probability of treatment weighting

The median period between surgery and full oral feeding was 1.1 months (IQR, 0.8–2.4) in the CSE group, whereas it was 1.5 months (IQR, 0.6–5.7) in the non-CSE group. After IPTW, the median period was 1.0 month (IQR, 1.0–2.0) in the CSE group and 2.0 months (IQR, 1.0–6.0) in the non-CSE group (Fig. 2). Full oral feeding was achieved by all patients in the CSE group within 6 months after surgery, whereas 9 of the 31 (29%) children in the non-CSE group remained dependent on tube feeding at 6 months after surgery. After IPTW, CSE was associated with a shorter period between surgery and full oral feeding [weight hazard ratio (wHR), 2.26; 95% CI, 1.21 to 4.24; Table 4]. These results were consistent with Cox regression adjusted for the same set of confounders (adjusted hazard ratio (aHR), 2.14; 95% CI, 1.02 to 4.48; Table 4).

**Table 4:** Associations of clinical swallowing evaluation with the period between surgery and full oral feeding and growth measures at 2–3 years of age

Outcomes	*N*	Parameters		
Full oral feeding		Incidence (per 100 person-month)	aHRa (95% CI)	wHRa (95% CI)
Non-CSE group	31	3.9	1.0 (ref)	1.0 (ref)
CSE group	19	4.2	2.14 (1.02, 4.48)	2.26 (1.21, 4.24)
Stunting at 2–3 y of age		Cases (%)a	aORa (95% CI)	wORa (95% CI)
Non-CSE group	15	6 (40.0)	1.0 (ref)	1.0 (ref)
CSE group	16	2 (12.5)	0.18 (0.02, 1.37)	0.20 (0.03, 1.44)
Underweight at 2–3 y of age		Cases (%)a	aORa (95% CI)	wORa (95% CI)
Non-CSE group	15	5 (33.3)	1.0 (ref)	1.0 (ref)
CSE group	16	1 (6.3)	0.07 (0.002, 2.70)	0.12 (0.01, 1.46)

The follow-up time for incidence was started at the date of surgery, and ended at the date of full oral feeding or lost to follow-up

*aHR* adjusted hazard ratio, *aOR* adjusted odds ratio, *wHR* weighted hazard ratio, *wOR* weighted odds ratio

^a^The same set of confounders was adjusted for or used for inverse probability weighting, including age at surgery, sex, place of residence, weight before surgery, and distance to the blind pouch of the esophagus

**Fig. 3:**
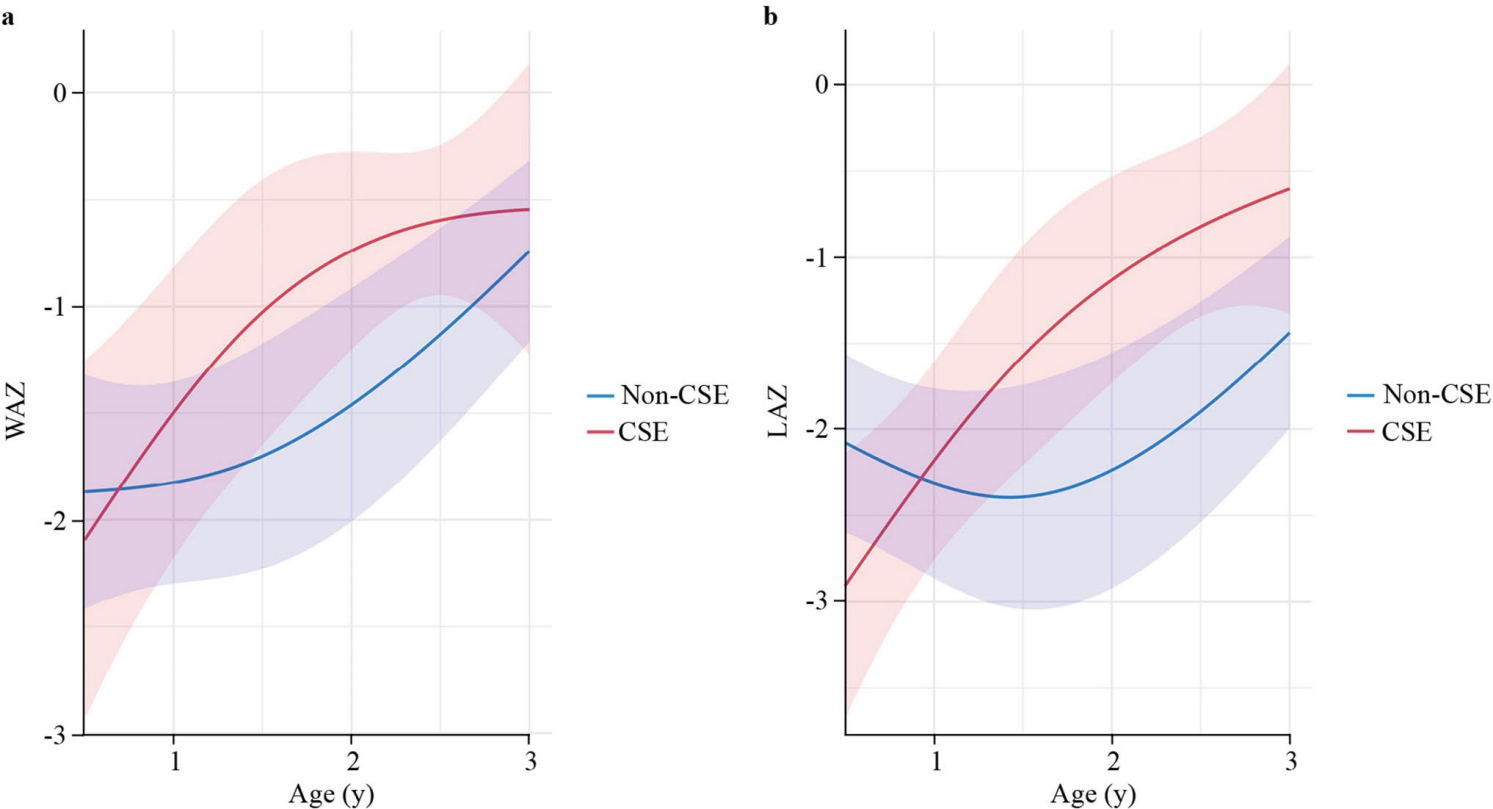
Estimated growth curves for WAZ (**a**) and LAZ (**b**) in CSE and non-CSE groups. *WAZ* weight-for-age *Z* score, *LAZ* length-for-age *Z* score, *CSE* clinical swallow evaluation

The growth curves for LAZ were significantly different between the CSE group and the non-CSE group (*P* = 0.001). After IPTW, the average LAZ in the CSE group decreased at 6 months of age, surpassed that in the non-CSE group, and increased continuously towards the population-average level (Fig. 3). Specifically, the estimated LAZ at two years of age was −1.13 (95% CI, −1.73 to −0.53; Supplementary Table 2) in the CSE group, compared to −2.24 (95% CI, −2.92 to −1.55) in the non-CSE group. Additionally, CSE was marginally associated with a lower incidence of stunting at 2–3 years of age [12.5% versus 40.0%; weighted odds ratio (wOR), 0.20; 95% CI, 0.03 to 1.44; *P* = 0.11; Table 4]. The growth curves for weight differed between the CSE group and the non-CSE group, with marginal significance (*P* = 0.10). Starting at a similar average level at 6 months after surgery, the WAZ increased at a greater rate in the CSE group than in the non-CSE group at two years after surgery. Specifically, the estimated WAZ at 2 years of age was −0.74 (95% CI, −1.20 to −0.28; Table S2) in the CSE group, compared to −1.46 (95% CI, −2.01 to −0.91) in the non-CSE group. Additionally, the CSE group had a lower incidence of underweight at 2–3 years of age than the non-CSE group (6.3% versus 33.3%; wOR, 0.12; 95% CI, 0.01 to 1.46; *P* = 0.09; Table 4).

In the CSE group, only 4 (21.1%) children developed pneumonia after surgery, compared to 17 (54.8%) children in the non-CSE group. However, the IPTW analysis revealed a risk estimate above unity (wOR, 0.28; 95% CI, 0.05 to 1.45; *P* = 0.13).

## Discussion

In our study, we observed that CSE was associated with a shorter time to achieve full oral feeding after surgery. Children in the CSE group exhibited a more favorable growth pattern in early life. In addition, the incidence of postoperative pneumonia was lower in the CSE group than in the non-CSE group. Our findings suggest that CSE might improve the growth status of children who undergo surgery for LGEA.

Surgical anastomosis for LGEA is generally performed within months after birth, a critical period for the development of swallowing function and growth in children. Previous studies have shown that swallowing dysfunction is a common problem in children with LGEA due to prematurity, anatomical abnormalities, and prolonged parenteral or enteral tube feeding [22, 23], which can affect their growth [24, 25]. However, due to the low incidence of LGEA, evidence of the best practice for the perioperative management of LGEA is limited [6, 17, 26–28]. Our study adds to the existing knowledge by demonstrating that CSE, a highly feasible method, may improve swallowing function and support better growth and development in children after surgery for LGEA.

CSE aims to improve oral feeding through tailored strategies for children with LGEA. CSE considers the developmental characteristics of children with LGEA and provides individualized treatment plans based on the results of oral peripheral examinations, oral feeding skill assessments, and treatment tests. In our approach to CSE for children with LGEA, we adopt cue-based feeding, which involves (1) using nonnutritive sucking to stimulate feeding behavior; (2) systematic behavior assessments to observe and identify infants’ feeding preparation clues, and (3) developing appropriate responses to help initiate oral feeding early in infants with LGEA [6, 7, 29, 30]. Evidence indicates that offering pleasant oral experiences is an important component of non-medical treatment, while maintaining oral and swallowing skills could minimize oral sensory abnormalities [31, 32]. Moreover, CSE can be used to guide meal arrangements, feeding skills, and parent‒child interactions, ensuring safe feeding strategies and helping children with LGEA achieve standard feeding expectations. The CSE consensus emphasizes the role of caregivers and the environment of the feeding process, integrating the concept of “making parents’ participation and training an important part of nursing”. Research indicates that when anxious about their child’s feeding problems, caregivers often resort to inappropriate measures, which can exacerbate feeding problems [33]. A study demonstrated that intervention approaches addressing feeding difficulties yielded positive results when administered according to a protocol by experienced therapists and complemented with parent education [34]. The findings proposed that parent training played a pivotal role in facilitating successful intervention outcomes. These factors may explain why the CSE group had a shorter time to complete oral feeding than the control group. Even after transitioning to oral feeding, regular CSE follow-up is still required for children with LGEA to ensure they meet feeding milestones. Moreover, given that dysphagia is a prevalent issue among patients with other types of esophageal atresia, implementing CSE in these cases may warrant further investigation.

Swallowing dysfunction in early life may impair children’s growth [24, 25]. The growth curves for WAZ and LAZ in our study showed that the length and weight of children with LGEA were below the average levels in early life. Despite starting at lower levels, the length and weight of children in the CSE group caught up with those of the children in the non-CSE group during the follow-up. In the CSE group, the WAZ and LAZ increased more rapidly around 1 year of age, highlighting the effectiveness and importance of early intervention. Previous evidence has also shown that, after the age of one year, the ability to swallow solid food is the main factor affecting these children’s growth and development [35].

Lung diseases, mainly pneumonia, are common complications in children with esophageal atresia [10, 13, 36]. In children with LGEA, the reported incidence of lung diseases can reach 80%, possibly due to delayed anastomosis [37]. These lung diseases could be a result of aspiration disorders, which can also affect the rhythm of sucking, swallowing, and breathing, leading to respiratory restrictions, decreased gas exchange, poor feeding efficiency, and unsafe oral feeding [38]. In our study, the incidence of pneumonia in the CSE group (21.1%; 4 of 19) was substantially lower than that in the non-CSE group (54.8%; 17 of 31). The standard CSE procedure includes screening and identifying risk factors. When swallowing and breathing abnormalities are present, infants are referred to a respiratory specialist promptly, and caregivers are provided training on respiratory care during the nursing process to reduce the frequency and severity of pulmonary diseases. Since eating disorders in children are significantly related to chronic respiratory symptoms [39], regular CSE follow-up is conducive to the realization of a child- and family-centered medical model [40] and fosters effective collaboration among multidisciplinary teams [41]. In general, the basic principles of safe feeding, timely referrals after screening, and respiratory home care are likely to be the main factors contributing to the reduced incidence of pneumonia in the CSE group.

Our study has the following strengths. First, we used a historical control group to reduce potential heterogeneity among patients and surgical procedures. Notably, as an indicator of surgical level, the rate of postoperative complications was consistent with international reports [37, 42, 43]. Furthermore, we observed no significant differences in the rate of postoperative complications (except for pneumonia) between the CSE and non-CSE groups, suggesting comparable surgical levels during the study period (Supplementary Table 6). Second, we applied a propensity score-based approach to further balance baseline characteristics between the groups. Lastly, we provided longitudinal data with multiple follow-ups to show the growth curve in early life.

Our study has the following limitations. First, the sample size was relatively small, resulting in relatively low statistical power. However, considering the low incidence of LGEA, our study included a relatively large sample of patients with LGEA [6, 27, 28]. Further researchers with larger sample sizes are required to validate our findings. Second, our study focused on a limited set of outcomes. Future research should examine additional outcomes such as caregiver confidence in oral feeding and the child’s proficiency with age-appropriate food textures. The assessment of children’s neurodevelopmental levels should also be considered, as growth and development were the basis of this study and are highly correlated with neurodevelopment outcomes [44–46]. Third, the follow-up only included data collected before three years of age. Since growth trajectories at two years of age are highly predictive of later growth and development [47, 48], further research should explore the impact of CSE on long-term growth and development. Fourth, while videofluoroscopic evaluation of swallowing (VFS) and fiber optic evaluation of swallowing (FFES) provide objective measurements of swallow functions, these evaluations were not routinely conducted on the patients included in this study, mainly due to the challenges in obtaining cooperation from young children. Instead, we used cervical auscultation in our study to preliminarily screen for risk factors such as impaired swallow function, although this method cannot accurately diagnose pharyngeal dysphagia. This highlights the need to develop methods for carrying out objective swallowing evaluations in young children in the future. Fifth, all CSE procedures in this study were conducted by a single evaluator. While this reduced heterogeneity, it could also introduce bias and impact the generalizability of the findings. Nevertheless, consistent findings in objectively measured growth outcomes supported the main results. Future studies should explore the efficacy of the CSE procedure across different settings.

## Supplementary Information

Below is the link to the electronic supplementary material.

Supplementary file1 (DOCX 391 KB)

Supplementary file2 (DOCX 1284 KB)

## Data Availability

The datasets used and analyzed during the current study are available from the corresponding author on reasonable request.
