# Supplementary material for: Impact of serial clinical swallow evaluations and feeding interventions on growth and feeding outcomes in children with long-gap esophageal atresia after anastomosis: a retrospective cohort study
Source: World J Pediatr. 2024 Nov 15;20(12):1293–305. doi: 10.1007/s12519-024-00850-x (PMC11634968; doi:10.1007/s12519-024-00850-x)
Supplement: Supplementary file 1 — Supplementary file1 (DOCX 391 KB) [file 12519_2024_850_MOESM1_ESM.docx]

**Impact of serial clinical swallow evaluations and feeding interventions on growth and feeding outcomes in children with long-gap esophageal atresia after anastomosis: a retrospective cohort study**

**Supplementary Material**

**Table of Contents**

[Table S1. Estimated odds ratios for covariates in calculating the propensity score. 2](#_Toc170836529)

[Table S2. Means of estimated weight-for-age z-score and length-for-age z-score by age. 3](#_Toc170836530)

[Table S3. Perioral/cerebral neurological examination (Age to be taken into account for application). 4](#_Toc170836531)

[Table S4. Developmental milestones scale for children's eating skills. 5](#_Toc170836532)

[Table S5. Examination of the external and internal structures of the oral cavity 13](#_Toc170836533)

[Table S6. Association between clinical swallowing evaluation and postsurgical complications. 14](#_Toc170836534)

[Figure S1. The whole process of LGEA patients’ management and treatment in CSE group 15](#_Toc170836535)

Figure S2. Propensity score distributional overlap A)before and B)after propensity score weighting. 16

Detailed information about the developmental-behavioral pediatrician who performed CSE in our study 17

# Table S1. Estimated odds ratios for covariates in calculating the propensity score.

| Covariates | OR (95% CI) |
| --- | --- |
| Distance from the blind pouch of the esophagus(cm) | 2.36(1.30,4.29) |
| Sex:Male | 1.46(0.36,5.92) |
| Place of residence:Rural or suburban | 1.06(0.38,2.93) |
| Weight before surgery(kg) | 0.42(0.19,0.93) |
| Age at surgery(days) | 1.01(1.00,1.03) |

OR, odds ratio.

# Table S2. Means of estimated weight-for-age z-score and length-for-age z-score by age.

|  | WAZ (95%CI) | | LAZ (95%CI) | |
| --- | --- | --- | --- | --- |
| Age, years | No CSE (n=31) | CSE (n=19) | No CSE (n=31) | CSE (n=19) |
| 0.5 | -1.87 (-2.42,-1.32) | -2.09 (-2.93,-1.26) | -2.08 (-2.60,-1.56) | -2.91 (-3.68,-2.14) |
| 0.75 | -1.85 (-2.34,-1.37) | -1.79 (-2.52,-1.05) | -2.21 (-2.72,-1.69) | -2.54 (-3.16,-1.92) |
| 1 | -1.82 (-2.30,-1.35) | -1.5 (-2.18,-0.82) | -2.31 (-2.86,-1.76) | -2.18 (-2.76,-1.61) |
| 1.5 | -1.7 (-2.23,-1.17) | -1.03 (-1.65,-0.41) | -2.39 (-3.04,-1.74) | -1.57 (-2.21,-0.94) |
| 2 | -1.46 (-2.01,-0.91) | -0.74 (-1.20,-0.28) | -2.24 (-2.92,-1.55) | -1.13 (-1.73,-0.53) |
| 2.5 | -1.13 (-1.63,-0.63) | -0.6 (-0.95,-0.24) | -1.90 (-2.54,-1.26) | -0.82 (-1.34,-0.30) |
| 3 | -0.74 (-1.16,-0.32) | -0.55 (-1.22,0.13) | -1.44 (-1.99,-0.88) | -0.60 (-1.32,0.12) |

WAZ, weight-for-age z-score; LAZ,length-for-age z-score;CSE,clinical swallow evaluations.

# Table S3. Perioral/cerebral neurological examination (Age to be taken into account for application).

| CN-V: Trigeminal Nerve | CN-Ⅶ: Facial nerve | CN-X: Vagus nerve | CN-XII: Hypoglossal nerve |
| --- | --- | --- | --- |
| - Chin open/close - Alternate on/off - Resistance to jaw closure | - Facial symmetry - Lip closure - Labial retraction - Pout (express anger or displeasure) - Alternate retraction and pouting - Intraoral pressure - Sip one's lips - Illustrate the teeth (of a tool) - Nip - Resist opening your eyes. | - Palate lifting - Vomiting - Tone | - Protruding tongue - Lift your tongue up and touch your upper lip. - Depressed tongue - External lateral motion touching left/right lip corners - Alternating external lateral movements - Lick one's lips - Smack one's lips - Internal Side Motion |

References:

1. Zulin Dou (2009) Assessing and Treating Dysphagia. People’s Medical Publishing House, Beijing

2. Kelly D. Hall (2001) Pediatric Dysphagia Resource Guide. Singular/Thomson Learning, San Diego, CA

# Table S4. Developmental Milestones Scale for Children's Eating Skills.

Assessment of eating skills includes motor development related to eating skills, level of development of the mandibular, tongue and lip/buccal muscles

| **Age**  **(mouths)** | **Motor Development Milestones**  **(feeding-related motor development)** | | **Developmental milestones of the lower jaw** | **Developmental milestones of the tongue** | **Lip/Buccal Developmental Milestones** |
| --- | --- | --- | --- | --- | --- |
| 0-1 | -Reflex movements of the limbs  -Lift head while on stomach，but cannot support head when held upright | -Sucking on finger put near infant’s mouth | -Phasic bite  -Minimal control of gradation | -Thinly contoured tongue  -Tongue and jaw word in unison  -Restricted movement due to large size compared to other structures  -Gag | -Pads of fatty tissue surrounded by cheek muscle that is not used  -Pursed for  -Due to mechanical movement of jaw, lips make contact  - Rooting |
| 1-2 | -Moves arm smoothly in a circle  -Swipes at objects  -Holds head up briefly while on stomach  -Raises head while sitting supports but head bobs | -Able to bring to mouth when lying down or sitting up | -Phasic bite persists  -Movement same as newborn | -Extension and retraction during sucking  -Tongue remains in mouth at rest ,may protrude past gums when swallowing | -Rooting strong  -Although some separation may be noted, lips typically move in unison with other facial structures |
|  | **3 months**  -Voluntary body control  -Lifts head and chest while prone  -Holds head up with minimum bobbing while sitting supports  -Reaches and grasps  -Keeps hands open frequently  **4 months**  -Head turns in all direction whether prone and seated  -Head normally held at midline and aligned with trunk when sitting supported  -Grasps small objects put into hand  -Brings objects to mouth  **5 months**  -Sits supported for up to 30 minutes  -Rolls from stomach to back  -Can be easily pulled to stand  -Swaps objects from hand to hand | -Baby is beginning to sit up  -Gaining head, trunk control  -Able to bring hand to mouth while object is in hand | -Phasic bite diminishing  -Improved head control possibly affects stabilization of jaw | -Relaxed appearance  -Separation of movement within the front, mid, and back of tongue  -Protrudes tongue while swallowing  -Gag elicited on half to one third back of tongue  -5 mouths: inhibition of tongue movement increased | -Rooting diminishes  -Reduction of sucking pads  -Development of facial muscles  -Control of central portion of lips  -Refinement of lower lip stability and upper lip activity  -Increased lip and cheek activity while sucking |
| 6-9 month | **6 months**  -Shoulder and head more stable  -Turns head freely  -Sits straight slightly supported or in a chair  -Balances well  -Reaches with one arm, grasps, and brings to mouth  -Turns and twists in all directions  -Creeps  **7 months**  -Holds without palm  -Transfers objects from hand to hand  -Cuts first tooth  -Pushes up on hands and knees, rocks  **8 months**  -Manipulates objects to explore  -Pulls up to stand but needs help to get down  -Crawls  **9 months**  -Stands alone briefly  -Gets down alone  -Cruises  -Sits unsupported  -Gets into and out of sitting position alone  -Explores with index finger | -Trunk stability allows for independent jaw, tongue movement  -Independent sitting  -Pincer grasp  -Extended reach  -Holds bottle  -Removes and replaces bottle | -Phasic bite extinguished  -Jaw becomes more stabilized allowing for movement in smaller ranges  -Lateral with slight diagonal movement of jaw | -Variety of actions emerging  -May flatten, spread, groove tongue  -Up/down movement during munching  -Gag diminished in strength  -Tongue sensitive enough to detect which foods can or cannot be mashed  -8-9 months: Child able to lateralized food | -Rooting extinguished  -6 months: Upper of lower lip draws in slightly and may see cheeks tighten; If child loses liquid, only at beginning or end of feeding  -Lower lip becomes active stabilizer  -Active use of lip corners and musculature around lips  -Able to keep bolus in between molars with the help of the lips and cheeks |
| 10-12 | **10 months**  -Crawls with bilateral leg-arm opposition.  -Sits from standing position.  -More fine control of hand movement.  **11 months**  -Stands alone  -Gets up from all fours position by pushing up  -Climbs up stairs  **12 months**  -Stand alone  -Pushes to stand from squat  -Climbs up and down stairs  -Uses a crayon  -Releases objects willfully  -Takes first steps with support | -Fine motor skills develop | -Emerging sustained controlled pressure on softer foods  -Controlled opening/closing(improving jaw grading ability)  -May see early emergence of circular rotary action as precision and control of jaw movements improve | -Uses all muscles to shape tongue  -Emergence of all ranges of angles of movement  -Improved precision, combinations, and consistency of movement patterns | -Both upper and lower lips may draw in independently  -Active use of lips and cheeks on solids  -Lower lip draws in to be cleaned by upper incisors or gums  -Child no longer pockets food and rarely loses food  -Drooling is rare |
| 13-24 | **15 months**  -Unceasing activity  -Walks with rapid run-like gait  -Walks a few steps backward and sideways  -Carries objects in both hands or waves while walking  -Throws ball with elbow extension  -Takes off shoes and socks  -Scribbles lines  **18 months**  -Walks up stairs with help  -Walks smoothly, runs stiffly  -Throws ball with whole arm  -Throws and catches without falling  -Jumps with both feet off floor  -Turns pages  -Scribbles in circles  -Has muscle control for toilet training  **21 months**  -Walks up and down stairs with help of railing or hand  -Jumps ,runs, throws, climbs  -Kicks large ball  -Squats to play  -Puts shoes on part way  -Unzips  -Fits things together, such as an easy puzzle  -Responds rhythmically to music with whole body  **24 months**  -Walk smoothly, watching feet  -Runs rhythmically, but unable to start or stop smoothly  -Walks up and down stairs alone without alternating feet  -Tip toes for a few steps  -Pushes tricycle |  | -12-14 months: will see emerging circular/rotary movement  -18-24months: will not need to turn head in direction of bite due to improved jaw grading skills | -Child learns to swallow with tongue tip elevation and stabilization at alveolar ridge  -Food texture will influence tongue movement patterns  -Tongue moves side to side across midline  -Tongue becomes the major cleaner for inside mouth  -12-24 months: jaw and tongue movements are independent of each other  -18-20 months: child may clean lips with tongue | -Continuation of sustained control of lip pressure and lip movements while tongue and jaw are moving( separation of control)  -Corners of lips may draw into help control placement and assist with movement |
| 24 + | -Distinguishes between finger and spoon foods  -Highly mobile  -Can walk up stair without hand being held  -Can jump from bottom step  -Can jump in place  -Can throw a ball  -Continues to refine movements | -Child can use a straw  -Holds small glass in one hand, replace glass without dropping  -Uses spoon correctly but with some spilling  -Begins to use fork, holds it in fist | -Uses sucking patterns and active internal jaw stabilization without biting edge of cup  -Internal stabilization occurs most of the time during drinking sequences of more than two sucks  -Slight up-down jaw motions or holding edge of cup with teeth may also occur  -Slight lateral movements of jaw may occur when sucking soft solid or pureed foods from spoon  -Uses controlled, sustained bite while keeping head at midline when food presented for biting on both sides of mouth  -Is able to grade opening of jaw when biting foods of various thicknesses  -Jaw movement in chewing continues to be mixture of nonstereotypic and diagonal rotary movements  -Circular rotary movements occur when transferring food across midline form one side of mouth to other | -Used on a free, sweeping motion to clean food from upper or lower lips  -Tongue elevation and depression are independent of jaw movement  -Skillful tongue tip action may be present  -Uses tongue tip elevation for swallowing  -Can transfer food rapidly and skillfully from center to side, from side to center, and from side to side across midline  -No extension-retraction movements occur, even with difficult food transfers | -Easy lip closure, with no loss of liquid during drinking or when the cup is removed from the lips  -Swallows with no loss of saliva or food  -Swallows solid foods, including those with a combination of textures, with easy lip closure as needed  -Adequate lip movement during chewing  -Can keep lips closed during chewing, but dose so only when needed to retain the food |

References:

1. Zulin Dou (2009) Assessing and Treating Dysphagia. People’s Medical Publishing House, Beijing

2. Kelly D. Hall (2001) Pediatric Dysphagia Resource Guide. Singular/Thomson Learning, San Diego, CA

# Table S5. Examination of the external and internal structures of the oral cavity

Method of recording: + Normal; - Of concern; ? Don't know/difficult to confirm; Pharynx needs to record tonsil size.

| framework | head | ear | eye | mouth | lower jaw | lip | tooth | hard palate | latch | soft palate | pharynx | Larynx/vocal cords |
| --- | --- | --- | --- | --- | --- | --- | --- | --- | --- | --- | --- | --- |
| magnitude |  |  |  |  |  |  |  |  |  |  |  |  |
| symmetry |  |  |  |  |  |  |  |  |  |  |  |  |
| placement |  |  |  |  |  |  |  |  |  |  |  |  |
| position |  |  |  |  |  |  |  |  |  |  |  |  |
| strain |  |  |  |  |  |  |  |  |  |  |  |  |

References:

1. Zulin Dou (2009) Assessing and Treating Dysphagia. People’s Medical Publishing House, Beijing

2. Kelly D. Hall (2001) Pediatric Dysphagia Resource Guide. Singular/Thomson Learning, San Diego, CA

Table S6. Association between clinical swallowing evaluation and postsurgical complications.

| **Complication[n(%)]** | **CSE group (n=19)** | **Non-CSE group (n=31)** | **wOR***  **(95% CI)** | **P value** |
| --- | --- | --- | --- | --- |
| **Pneumonia** | 4 (21.1) | 17 (54.8) | 0.28 (0.05 , 1.45) | 0.13 |
| **Other complications^***^** |  |  |  |  |
| Anastomotic stenosis | 14 (73.7) | 20 (64.5) | 0.28 (0.11, 2.41) | 0.40 |
| Reflux esophagitis | 12 (63.2) | 13 (41.9) | 1.47 (0.33, 6.50) | 0.60 |
| Hiatal hernia | 5 (26.3) | 6 (19.4) | 0.68 (0.13, 3.54) | 0.64 |
| Anastomotic leak | 2 (10.5) | 3 (9.7) | —** | —** |
| Tracheo-esophageal fistula | 1 (5.3) | 4 (12.9) | —** | —** |
| Tracheal stenosis | 1 (5.3) | 2 (6.5) | —** | —** |

CSE, clinical swallow evaluation; wOR, weighted odds ratio.

* The following confounders were used for inverse probability weighting: age at surgery, sex, place of residence, weight before surgery and distance from the blind pouch of the esophagus.

** Insufficient case numbers.

*** We hypothesized that the comparable rates of these complications between the two groups might indicate that the observed associations in the study were less likely to be fully explained by the differences in surgical techniques.


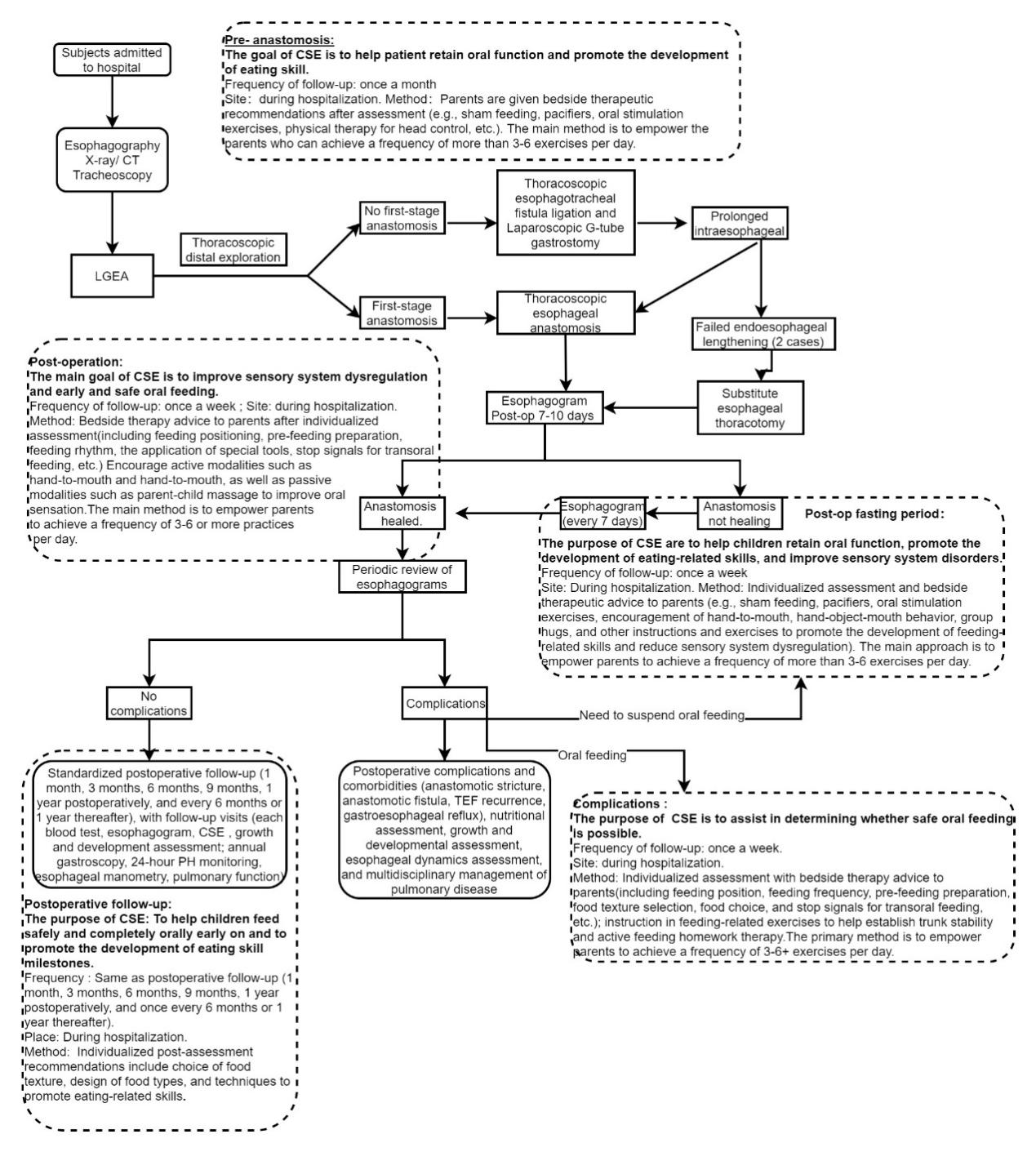


**Figure S1. The whole process of LGEA patients’ management and treatment in CSE group.** Non-CSE group had no processes of dotted lines.


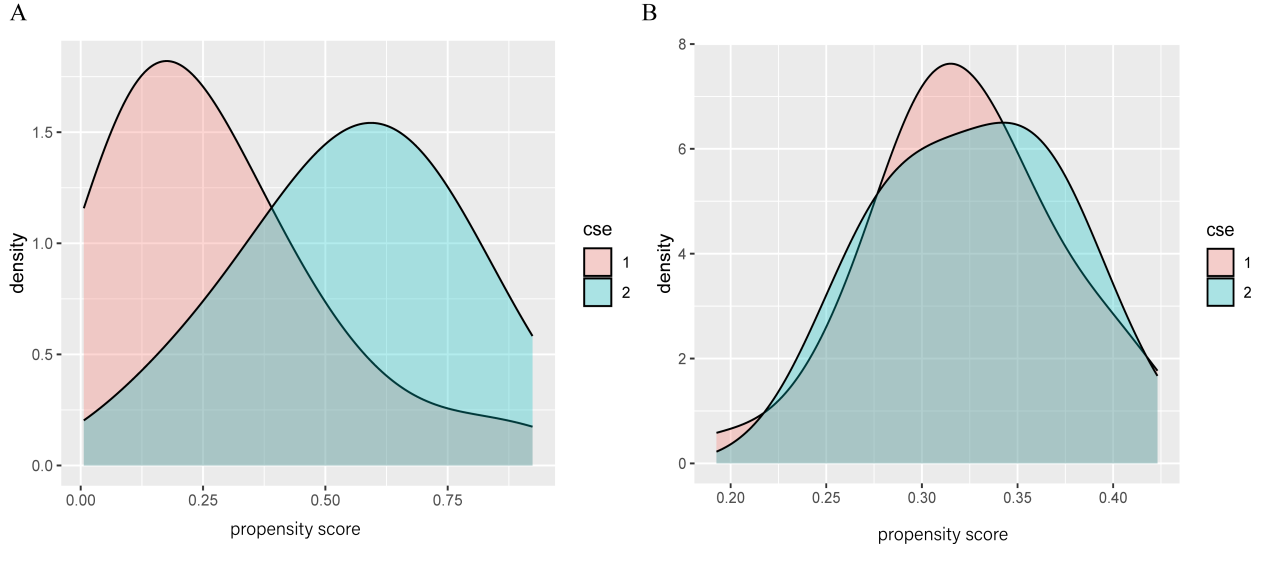


# Figure S2. Propensity score distributional overlap A)before and B)after propensity score weighting.

CSE, clinical swallow evaluations.

CSE=1, non-CSE group; CSE=2, CSE group.

Detailed information about the developmental-behavioral pediatrician who performed CSE in our study

A developmental-behavioral pediatrician generally refers to a specialist who hold certification in pediatrics and has additional subspecialty training in developmental-behavioral pediatrics. To be noted, an MD in China is an academic qualification (commonly, 5 years for a bachelor, 3 years for a master, and then 3 years for an MD). The practice of medicine generally requires a bachelor-level degree on clinical medicine and residency training. That is to say, a specialist could also hold a bachelor-level degree. In this study, all CSEs were performed by JL Wang, who holds a PhD in Pediatrics and has received training of special children's feeding in New Jersey Special Children's Hospital. Upon returning to China, she was integrated into the multidisciplinary team (MDT) for esophageal atresia.
