# Supplementary material for: Impact of serial clinical swallow evaluations and feeding interventions on growth and feeding outcomes in children with long-gap esophageal atresia after anastomosis: a retrospective cohort study
Source: World J Pediatr. 2024 Nov 15;20(12):1293–305. doi: 10.1007/s12519-024-00850-x (PMC11634968; doi:10.1007/s12519-024-00850-x)
Supplement: Supplementary file 2 — Supplementary file2 (DOCX 1284 KB) [file 12519_2024_850_MOESM2_ESM.docx]

**临床吞咽评估促进长段缺失型食管闭锁儿童早期发展**

**导读**

先天性食管闭锁是新生儿期消化道的一种严重的先天性畸形，当近、远端食管盲端相距超过3.5cm，即长段缺失型食管闭锁（LGEA），往往无法行一期食管端端吻合术，限制了生后立即进行食管重建，且在吻合术后还要通过非经口到经口喂养的挑战。临床吞咽评估包括评估前病例回顾、照顾者/儿童访谈、直接评估、筛查（危险信号）和咨询、建议以及治疗计划五个部分，助力家庭中心式多学科协作模式，促进LGEA患儿的早期发展。

**图文摘要**

**
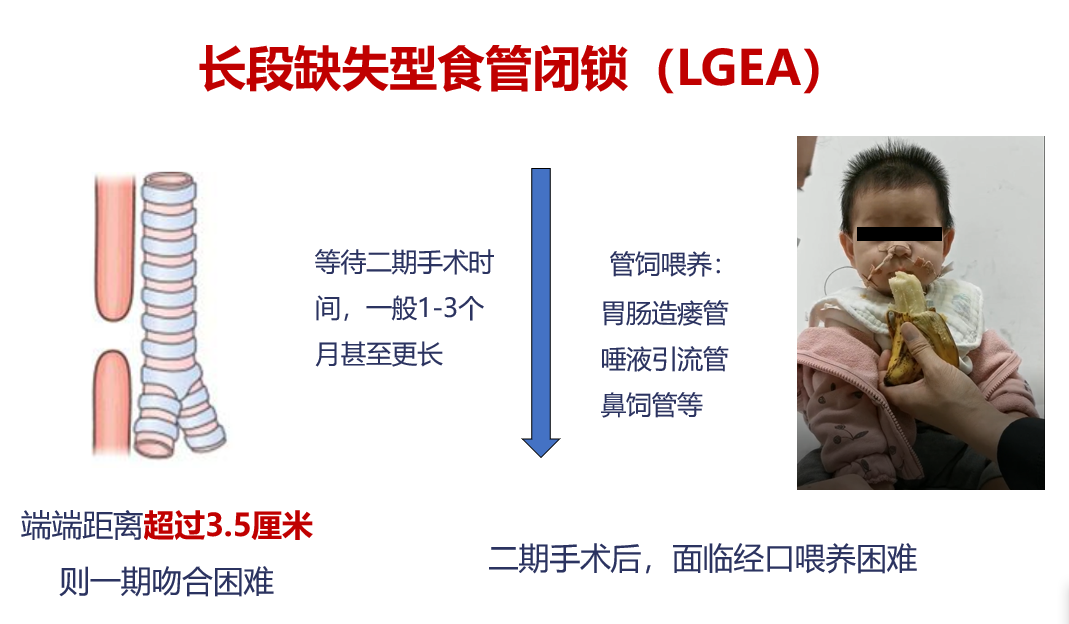
**

**图1 长段缺失型食管闭锁**

先天性食管闭锁发病率约1/(3000-4000)，其中LGEA约占10%。LGEA的特点是无法进行一期吻合，这对恢复食管的连续性构成了重大挑战，且术后并发症发生率高，包括短期胃肠道并发症，如吻合口狭窄/漏；短期呼吸并发症，如气胸、肺炎和肺不张；长期胃肠道结局，包括吞咽困难、反流和倾倒等；长期呼吸道结局，包括反复肺炎和反复吸入导致慢性肺部疾病，这些挑战多数发生在LGEA患儿的婴幼儿时期，影响他们的早期发育水平。LGEA管理和随访的复杂，提倡包括儿外科、消化专科、肺病专科、耳鼻喉科、营养咨询和言语治疗师等多学科协作，但仍面临非经口喂养到经口喂养的管理困难。本研究在多学科协作中纳入了临床吞咽评估技术（见表1），利用它的优势，包括根据LGEA患儿的病因、预后、饮食和治疗需求为照顾人员提供咨询和教育；制定喂养计划，包括术前提早进行假喂养，术后根据疾病状态和进食技能发育水平给予安全有效的饮食；制定治疗计划，通过体位指导、口内和口外按摩、行为干预策略提高饮食效率和进食技能；根据筛查危险信号及时进行转诊，包括仪器性吞咽评估或其他医疗转诊等，有利于建立以儿童为主导的家庭中心模式的多学科协作（如图2）。

**表1 临床吞咽评估流程（CSE）**

| **病例回顾** | | **根据医学、发育和喂养的经验，为孩子的表现提出可能的临床假设** |
| --- | --- | --- |
| **喂养表现** | **照顾者访谈**：喂养史和当前喂养状态。 | 确定影响标准喂养进展和期望的问题及障碍：弄清楚喂养困难的开始时间，是持续性还是急性；提供当前有关用餐时间安排、地点、具体策略、经口和肠内摄入的信息。 |
|  | **直接评估：**口腔外周检查，口腔喂养技能，治疗试验。 | 确定吞咽的结构和功能是否存在问题，识别技能病因；  提供用餐时间有关技能贡献、障碍和亲子互动的信息。 |
| **筛查/识别潜在危险**   1. 吞咽 2. 呼吸 3. 胃肠道/营养 4. 神经系统 5. 其他医疗 6. 发育 | | 确定仪器评估的必要性；  确定喂养困难的潜在危险因素。 |
| **咨询、推荐和治疗计划** | | 就病因、预后、饮食和治疗需求为照顾人员提供咨询/教育；  制定喂养计划（儿童安全有效地饮食）；  制定治疗计划（提高饮食和技能的推荐目标和策略）；  进行转诊（仪器性吞咽评估或其他医疗转诊）。 |

**
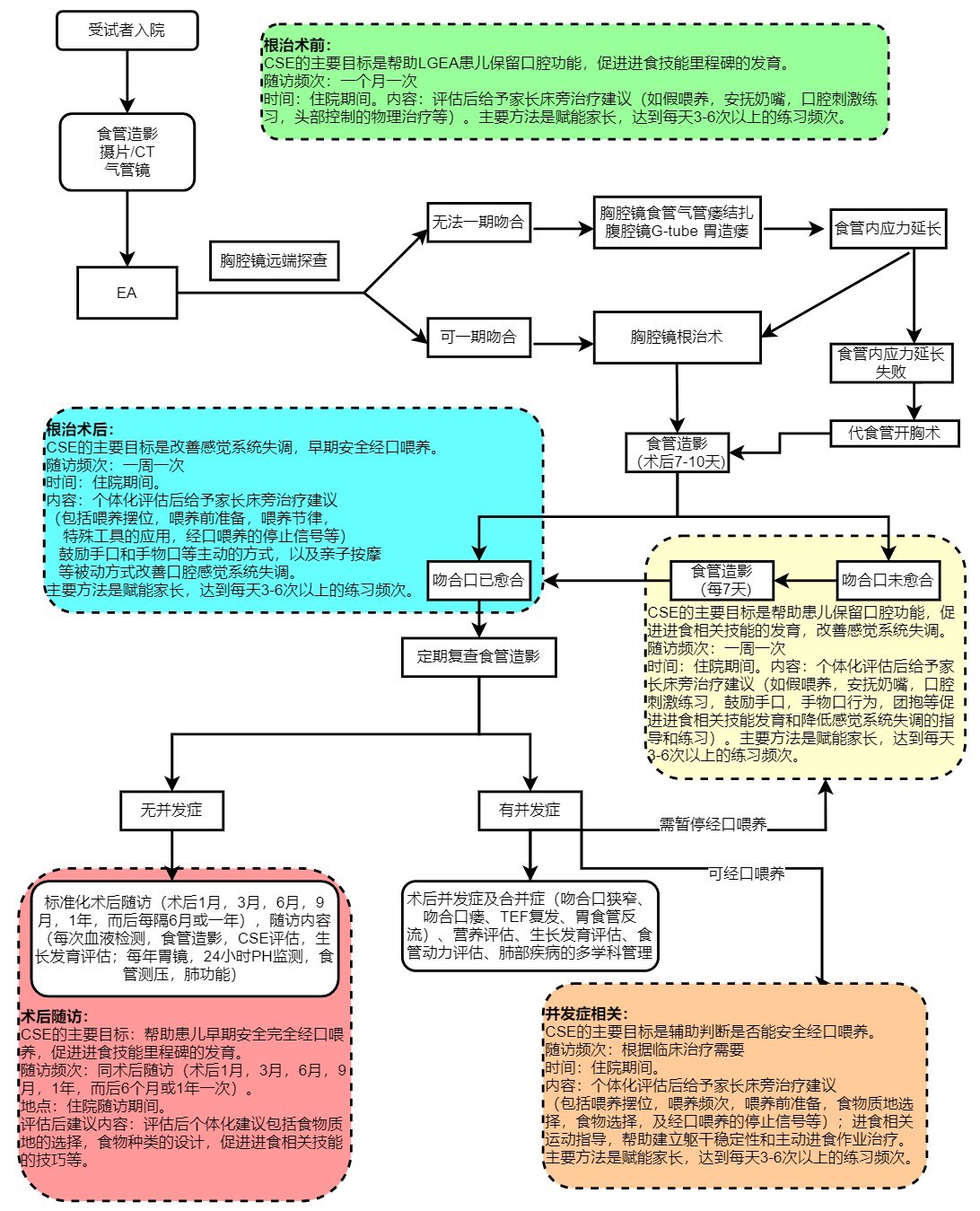
**

**图2 LGEA儿童队列随访流程图（CSE组包括彩图流程，对照组不包括彩图部分）**

**
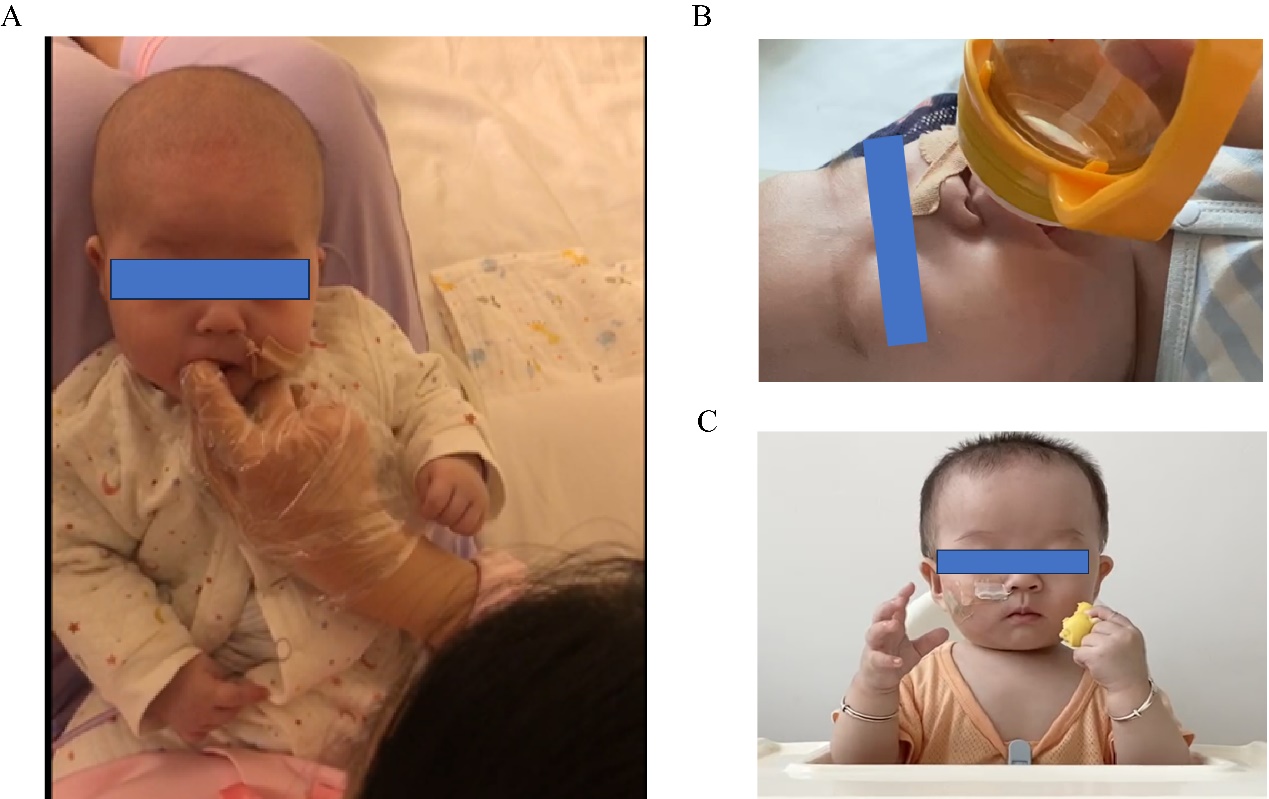
**

**图3 典型个案介绍**

某男，孕33周胎内B超提示胃泡未显示提示先天性食管闭锁。孕38周+3天，剖宫产，出生体重2900克。于出生当日行“腹腔镜下胃造瘘术+远端食管造影术”诊断为先天性食管闭锁I型。因食管两端间隔7.5厘米，吻合术行于7月龄23天。**术前CSE评估，**发现唾液引流管自自口腔，改为鼻腔入，减少口腔刺激；进食技能低下，通过假吸吮等口内训练，鼓励母亲与患儿面对面互动，抚触患儿口唇如图3A，降低感觉防御和改善口腔感觉功能；通过运动康复（根据发育年龄，帮助患儿实现了3月龄抬头，5月龄翻身），增加躯干的稳定性，以促进咽部和食管功能，为经口喂养做准备。因等待吻合术时间长，中间定期进行CSE，包括手口练习，手物口练习等，以不断改善口腔各部分功能，即唇颊，舌和下颌的独立动作，以及它们的协同性。**围手术期CSE评估**，包括在术前开始引入无空气奶瓶吸吮练习如图3B，为术后经口喂养做准备。**术后CSE评估，**包括建议保留胃造瘘，影像学检测术后食管功能（经口安全喂养的前提）确定食管结构完整，进行吞咽水练习让患儿感受真正的吞咽；在吞水熟练后（熟练吞咽5毫升水），开始特殊配方奶经口喂养。在影像学检测后3天后达到了完全经口喂养。后期继续定期CSE，帮助儿童引入固体食物如图3C，食物多样性，并指导回应性喂养，促进患儿的早期发育。

**研究结果**

回顾性队列研究。选取在上海交通大学医学院附属新华医院小儿外科2016 年 1 月～ 2023年12月内随访的LGEA队列随访儿童。其中2020年1月开始对新入组的LGEA儿童进行定期CSE评估，被列为CSE组，前期随访儿童列为对照组(见图2)。

1. **术后经口喂养的间隔时间更短如图4**

CSE组手术和完全口服喂养之间的中位时间为1.1个月（IQR，0.8-2.4），而非CSE组为1.5个月（IQ，0.6-5.7）。


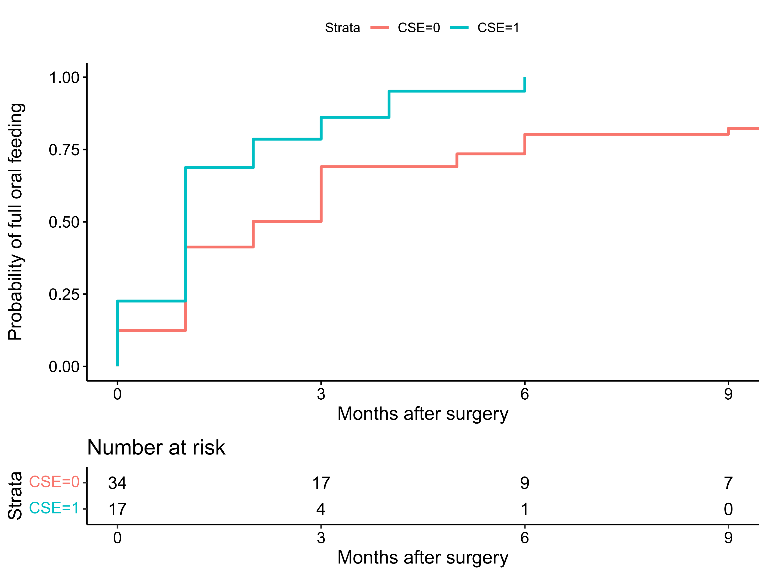


图4 两组术后完全经口喂养的概率比较

1. 1岁后体格生长曲线更优。

CSE组和非CSE组的LAZ生长曲线存在显著差异（P=0.001），如图5，


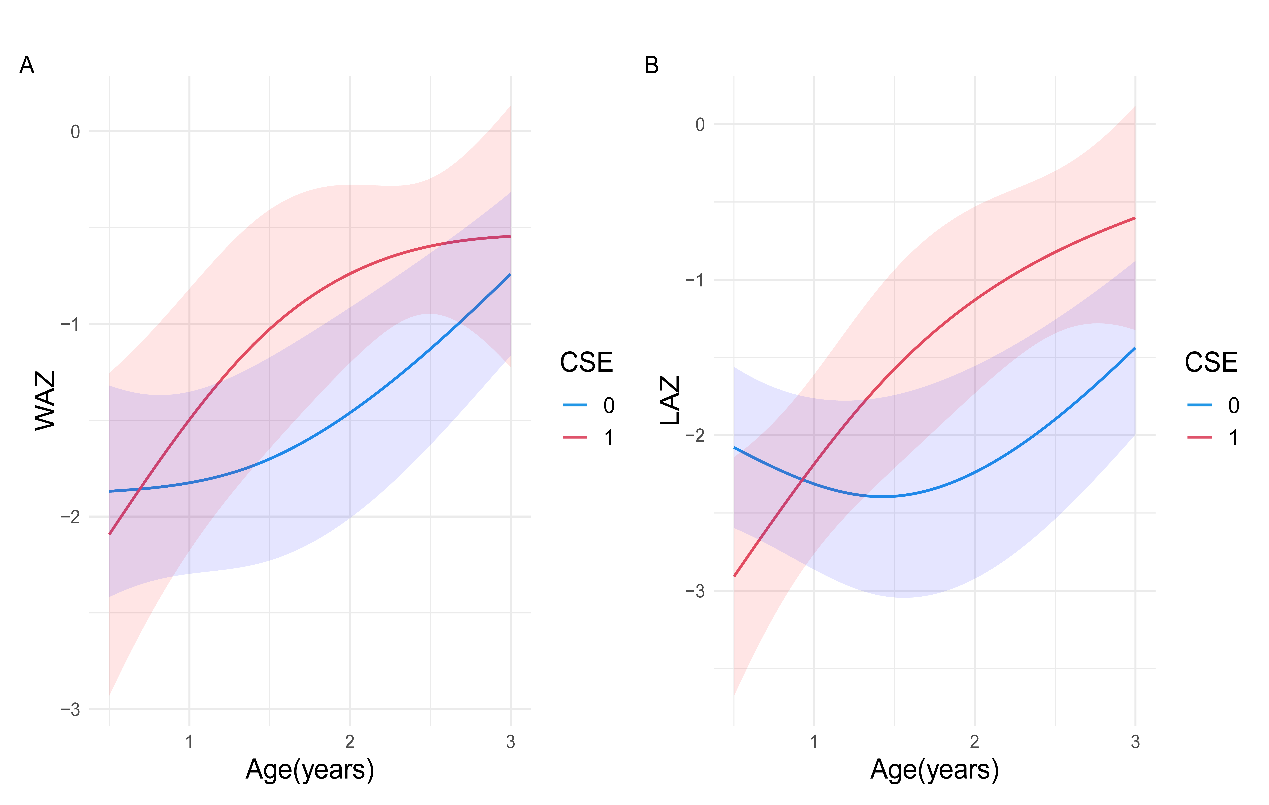


图5 两组术后WAZ和LAZ生长曲线图

**总结和展望**

本研究建议对LGEA儿童自新生儿期开始CSE随访，不仅有利于建立以儿童为主导的家庭中心式多学科协作模式，也有利于降低完全经口喂养间隔时间，改善1-3岁的体格追赶，从而促进他们的早期发育。
